# Prevalence of Latent Equid Herpesvirus Type 1 in Submandibular Lymph Nodes of Horses in Virginia

**DOI:** 10.3390/pathogens12060813

**Published:** 2023-06-07

**Authors:** Nadia Saklou, Scott Pleasant, Kevin Lahmers, Rebecca Funk

**Affiliations:** 1 Department of Biomedical Sciences and Pathobiology, Virginia-Maryland College of Veterinary Medicine, Virginia Tech, The University of Maryland, Blacksburg, VA 24060, USA; 2 Department of Large Animal Clinical Sciences, Virginia-Maryland College of Veterinary Medicine, The University of Maryland, Blacksburg, VA 24060, USA

**Keywords:** horse, equine herpesvirus 1, herpesvirus, latency, submandibular lymph nodes, PCR

## Abstract

Equine Herpesvirus type 1 (EHV-1) typically causes mild respiratory disease, but it can also cause late-term abortion, neonatal foal death and neurologic disease. Once a horse is infected, the virus concentrates to local lymphoid tissue, where it becomes latent. The virus can be reactivated during times of stress, which can lead to the initiation of devastating outbreaks. Understanding the carriage rate of latent EHV-1 in different geographic regions is essential for managing the disease. The objective of the current study was to estimate the prevalence of latent EHV-1 and compare the frequency of each variant in the submandibular lymph nodes of horses in Virginia. Sixty-three submandibular lymph nodes were collected post-partem from horses submitted to regional labs for necropsy, and qPCR was performed. All samples were negative for the *gB* gene of EHV-1. The results demonstrated a low apparent prevalence of latent EHV-1 DNA in submandibular lymph nodes in this population of horses in Virginia. Despite this, the mainstay for outbreak prevention and mitigation continues to focus on minimizing risks and using appropriate and diligent biosecurity.

## 1. Introduction

Equine Herpesvirus type 1 (EHV-1) is a double-stranded DNA virus that typically causes mild respiratory disease. However, viremia can lead to late-term abortion, neonatal foal death and neurologic disease (equine herpesvirus myeloencephalopathy or EHM) [1,2,3]. The virus is ubiquitous in equine populations and affects horses of every breed and discipline. Once a horse is infected, the virus localizes to trigeminal ganglia or lymphoid tissues for latent genome maintenance [4,5].

While the virus is latent, the animals are not infectious and are clinically normal. However, the virus can be reactivated during times of stress, such as transport or competition. The recrudescence of EHV-1 infections is sporadic but has also been attributed to the initiation of outbreaks, which have proven to be damaging for multiple facets of the equine industry. Recrudescence is likely responsible for the spread of EHV-1 to susceptible horses, resulting in outbreaks [6].

One of the most devastating EHV-1 outbreaks occurred in Europe, starting in February 2021 during an international show jumping competition. Ten countries were involved with at least 118 horses infected, 18 of which died. Equestrian events across Europe were halted until the middle of April 2021, having profound economic effects across the industry [7].

Considering the role that latency, and the reactivation thereof, plays in maintaining the virus within a population and initiating outbreaks, respectively, the mechanisms of recrudescence, including its environmental triggers, are of key importance for disease control. These facets of EHV-1 are currently poorly understood. Combined with a lack of feasibility of the ante mortem detection of latently infected horses, there is a profound paucity in well-rounded EHV-1 disease management strategies.

Understanding the carriage rate of latent EHV-1 in different regions is essential for the management of the disease. Currently, there are few studies regarding the prevalence of latent EHV-1 in horses, and none specifically in Virginia. The objective of the current study was to estimate the prevalence of latent EHV-1 and compare the frequency of each variant in the submandibular lymph nodes of horses in Virginia.

## 2. Materials and Methods

**Figure 1:**
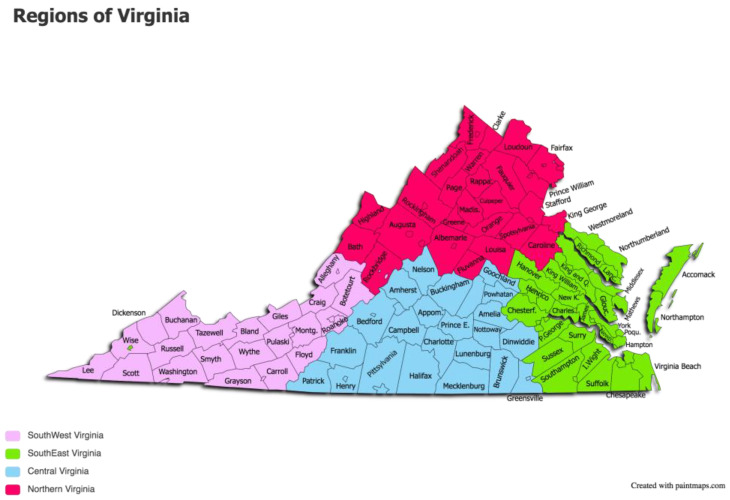
Regions of Virginia. These regions were predetermined based on the locations of 5 regional diagnostic laboratories. These are the areas from which horses were submitted to the laboratory for processing. The regions are indicated by each color, as noted in the legend. Not shown is the region of Outskirts of Virginia (OVA). This is the surrounding area of the state from where horses were received by the regional laboratories.

The study sample consisted of a total of 63 equids submitted for necropsy to diagnostic labs in 5 different pre-determined regions of Virginia from January 2020 to March 2021. The pre-determined regions of Virginia were Northern Virginia (NVA), Southwest Virginia (SWVA), Central Virginia, Southeast Virginia (SEVA), and the Outskirts (OVA) (see Figure 1). Inclusion criteria included horses and mules over the age of 2 years that did not have signs of acute respiratory or neurologic disease that could have been due to EHV-1.

### 2.1. SMLN Collection

Submandibular lymph nodes (SMLNs) were collected from each study animal during routine postmortem examination at each of 5 regional diagnostic laboratories in Virginia. The entirety of the SMLNs were retrieved from each study animal using sterile, disposable instruments. The samples were placed into sterile whirlpacks and immediately stored at −80 °C until further processing. Fresh, disposable instruments were used for sample collections from each animal to avoid cross-contamination. Sample collection procedures were approved by the Institutional Animal Care and Use Committee of Virginia-Maryland College of Veterinary Medicine prior to collection. 

### 2.2. DNA Isolation

DNA extraction was performed under a biosafety cabinet with sterile labware to reduce environmental contamination and DNA carryover between samples. DNA was extracted from all samples using a Qiagen DNeasy Blood and Tissue Kit (Qiagen, Valencia, CA, USA). Based on the manufacturer’s recommendations, a 25 mg piece of SMLN from each horse was placed in individual Petri dishes and minced. The minced tissue was transferred to a clean microcentrifuge tube containing Buffer ATL (Qiagen, Valencia, CA, USA) and 40 µL proteinase K, then digested overnight at 56 °C. A mixture of the tissue lysate, buffer AL and ethanol was pipetted into a DNeasy Mini spin column (Qiagen), then centrifuged for 1 min at 13,300 rpm. The supernatant was discarded, and Buffer AW1 (Qiagen) was added to the spin column, and then centrifuged again. The spin column was then washed with Buffer AW2 (Qiagen), which was centrifuged for 3 min. The DNA was eluted in 100 µL of Buffer AE (Qiagen, Valencia, CA, USA) via incubation at room temperature for 1 min before centrifugation for 1 min at 13,300 rpm. The DNA was stored at −20 °C until downstream processing took place.

### 2.3. Polymerase Chain Reaction

Each sample of purified nucleic acid underwent a PCR preamplification step as previously described [7]. Briefly, a reaction was performed using Advantage 2 Polymerase Mix (Takara Bio USA, San Jose, CA, USA) containing the target primers for the *gB* gene and the *ORF30* gene of EHV-1 in a 50 µL total volume. The primer mix was amplified in a thermocycler (Biometra, Gottingen, Germany) under the following conditions: 1 min at 94 °C, followed by 25 cycles of 15 s at 94 °C, 15 s at 55 °C and 45 s at 70 °C, followed by 5 min at 70 °C.

All samples were tested for the presence of the gB and 2 variants (A and G) of the DNA polymerase (*ORF30*) gene of EHV-1 as previously described [8]. In total, 9 of the 63 samples were also tested for the C variant of the *ORF30* gene as previously described [9]. Nine samples were also tested for the presence of the C variant of EHV-1. 

Four positive controls were used in this study as follows:

An SMLN from a horse with confirmed natural latent infection with the G_2254_ strain of EHV-1 was used as a positive control of SMLN; DNA from a horse with confirmed, natural infection with the C_2254_ strain of EHV-1; DNA from wild-type EHV-1 (National Veterinary Services Laboratory, Ames, IA, USA) as a control for A_2254_, and DNA from EHV-1 vaccine (Rhinomune, Boehringer Ingelheim Vetmedica, St. Joseph, MO, USA) as a control for G_2254_.

## 3. Results

Samples were received from a total of 62 horses and 1 mule from five pre-determined regions of Virginia: 17 from NVA, 30 from SWVA, 11 central, 1 SEVA, 4 OVA, and the rest of the samples were from an unknown region of Virginia. There were 35 geldings and 18 mares, and the remainder were undefined. The age of the horses and mule ranged from the age of 3 years to 25 years; 11 were 5–7 years, and 8 were over 20 years (median 12.0 years).

Several breeds were represented, most of which were quarter horses (17 horses), thoroughbreds (14), warmbloods (5) and unlisted (14), but these also included American paint horses (2), standardbreds (2) a saddlebred (1), a Percheron (1), a Morgan (1), a mustang (1), a Tennessee walking horse (1), ponies (2), a Gypsy Vanner (1), a Norwegian fjord (1), an Arabian (1), a Haflinger (1), a spotted saddle horse (1) and a mule (1).

The control positive SMLN consistently tested PCR positive for the *gB* gene of the EHV-1 and G variants of the *ORF30* with a Ct of 25.54 and 25.92, respectively. The A_2254_ and C_2254_ controls had a Ct of 26.08 and 25.90, respectively. All of the 63 SMLN samples were qPCR negative for the *gB* gene of EHV-1. In total, 63/63 were negative for the *ORF30* gene for the A, G and C variants with a 95% confidence interval of 0–5.75 [10]. 

## 4. Discussion

This study represents the first evaluation of the prevalence of EHV-1 latency in Virginia with an attempt to evaluate throughout the region. We were anticipating a frequency of detection somewhere between 3.3 percent and 54 percent based on studies by Pusterla and others [11] and Allen and others [12], respectively. In this current study, we found a low estimated prevalence. The Californian study, which was in a sample population of 147 horses, 4 mules and 3 donkeys, had low prevalence in submandibular lymph nodes as well [11]. Our findings were dissimilar to those of the study in Kentucky where they assessed the prevalence of EHV-1 in SMLN of 132 thoroughbred mares in Kentucky [12]. Although we did not find any positives in this population of horses, we were able to detect EHV-1 in samples that were confirmed positive. Furthermore, the confidence interval of our current study indicates a similar estimated prevalence in submandibular lymph nodes to the Californian study. This does not equate to the zero prevalence of EHV-1 in horses, as our findings are an estimate of the prevalence in horses in the study area. For instance, the confidence interval indicates the range that the prevalence might have been, given that we had zero detections out of 63 equids sampled. Additionally, we cannot rule out latency in other tissues, such as the retropharyngeal lymph nodes or trigeminal ganglia, that have been shown to carry EHV-1 in latency [13]. It is plausible that the overall prevalence of EHV-1 of this population of horses in Virginia is low; however, follow-up studies in a larger population of horses and multiple sampling sites is likely warranted to make a more accurate prevalence conclusion in this region. Based on a recent affinity study, samples from retropharyngeal lymph nodes, pharyngeal roofs and trigeminal ganglion should be included with submandibular lymph node samples for future prevalence studies [13]. Thus, the estimated prevalence of EHV-1 in, specifically, the SMLNs of horses in Virginia is low.

Differences in prevalence in this sample type could be related to the variability in disciplines or horse use across the country. For instance, in 2003, 39% of California’s horse population was used for showing or racing [14]. This is compared to a report from 2001 indicating that 24.5% of Virginia horses were used for showing or racing [15]. The population of horses used in the Allen paper were specifically thoroughbred broodmares. These horses are required to endure live cover breeding and thus are typically transported frequently and interact with horses from a wide geographic area. These discipline differences should be considered when looking at the variable prevalences reported.

While the lymph node sample size in this current study (25 mg) is small compared to the entire organ, the PCR results from the Pusterla 2010 study showed that EHV-1 is evenly distributed in the tissue during latency. Our detection method consisted of PCR pre-amplification followed by traditional qPCR, which appropriately detected EHV-1 in our positive control lymph node. This is also true considering our sample size of 25 mg compared to previous studies using 500 mg [11,12].

The low estimated prevalence in this population of horses indicates that the focus of outbreak mitigation should be on preventing exogenous infection with consistent vaccination, prompt diagnosis and diligent biosecurity. Current vaccine recommendations for EHV-1 are bi-annual intramuscular inoculations [16]. It is also prudent to encourage horse care-takers to be meticulous in their monitoring and timely in their outreach for veterinary intervention when early clinical signs are noted. In horses that develop EHV-1 infection and recover, care should be taken to abate the likelihood of recrudescence. This can be achieved by minimizing stress and providing prudent biosecurity, particularly with those horses; continuing every 6-month vaccination; and remaining diligent with monitoring and prompt diagnosis and appropriate treatment.

In conclusion, the results of the current study demonstrated a low estimated prevalence of latent EHV-1 DNA in, specifically, submandibular lymph nodes in this population of horses in Virginia. Despite this, the mainstay for outbreak prevention and mitigation continues to focus on minimizing risks and using appropriate and diligent biosecurity.

## Data Availability

All data is provided within this manuscript.
